# Penta­potassium μ-arsenato-bis­(hy­droxy­tetra­molybdate) dihydrate

**DOI:** 10.1107/S1600536810054176

**Published:** 2011-01-15

**Authors:** Hai-Hui Yu, Li Kong, Ji-Wen Cui, Hai-Tao Wang

**Affiliations:** a College of Chemical Engineering, Northeast Dianli University, Jilin 132012, People’s Republic of China; b Jilin Institute of Chemical Technology, Jilin 132012, People’s Republic of China; c College of Chemistry and Pharmacy, Jiamusi University, Jiamusi 154000, People’s Republic of China; d Chemical Engineering Department, Huizhou University, Huizhou 516001, People’s Republic of China

## Abstract

The title arsenatomolybdate, K_5_[Mo_8_O_24_(OH)_2_(AsO_4_)]·2H_2_O, which was obtained hydro­thermally, features an [AsMo_8_O_28_(OH)_2_]^5−^ anion, which is formed by two Mo_4_O_14_(OH) units that are linked by As in a sandwich-like fashion. The overall symmetry of the anion is *m*2*m*. The {Mo_4_O_14_(OH)} core is composed of two pairs of confacial biocta­hedral {Mo_2_O_9_} units with two μ_4_-O atoms which have been characterized as hydroxyl groups. The anions are further inter­connected by potassium cations, forming a three-dimensional network structure with the uncoordinated water mol­ecules occupying the channels. The structure is further stabilized by O—H⋯O hydrogen bonding.

## Related literature

For isotypic K_5-*x*_(NH_4_)_*x*_P[Mo_4_O_14_(OH)]_2_·2H_2_O (*x* = 2.43), see: Chen *et al.* (2009 ▶). For a general overview of polyoxometalates, see: Pope (1983 ▶). For the Mo/As/O system, see: Sun *et al.* (2007 ▶); He & Wang (1999 ▶). For bond-valence-sum calculatios, see: Brown (1981 ▶); Hsu & Wang (1997 ▶).

## Experimental

### 

#### Crystal data

K_5_[Mo_8_O_24_(OH)_2_(AsO_4_)]·2H_2_O
*M* *_r_* = 1553.95
Orthorhombic, 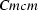

*a* = 8.3024 (12) Å
*b* = 23.008 (3) Å
*c* = 15.279 (2) Å
*V* = 2918.6 (7) Å^3^
*Z* = 4
Mo *K*α radiation
μ = 5.28 mm^−1^
*T* = 293 K
0.20 × 0.18 × 0.16 mm

#### Data collection

Rigaku R-AXIS RAPID diffractometer
Absorption correction: multi-scan (*ABSCOR*; Higashi, 1995 ▶) *T*_min_ = 0.418, *T*_max_ = 0.486
12346 measured reflections
1606 independent reflections
1216 reflections with *I* > 2σ(*I*)
*R*_int_ = 0.048

#### Refinement

*R*[*F* ^2^ > 2σ(*F* ^2^)] = 0.032
*wR*(*F* ^2^) = 0.098
*S* = 1.07
1606 reflections
133 parameters
Only H-atom coordinates refined
Δρ_max_ = 1.99 e Å^−3^
Δρ_min_ = −0.72 e Å^−3^

### 

Data collection: *RAPID-AUTO* (Rigaku, 1998 ▶); cell refinement: *RAPID-AUTO*; data reduction: *RAPID-AUTO*; program(s) used to solve structure: *SHELXS97* (Sheldrick, 2008 ▶); program(s) used to refine structure: *SHELXTL* (Sheldrick, 2008 ▶); molecular graphics: *DIAMOND* (Brandenburg, 2007 ▶); software used to prepare material for publication: *SHELXL97* (Sheldrick, 2008 ▶).

## Supplementary Material

Crystal structure: contains datablocks I, global. DOI: 10.1107/S1600536810054176/fi2098sup1.cif

Structure factors: contains datablocks I. DOI: 10.1107/S1600536810054176/fi2098Isup2.hkl

Additional supplementary materials:  crystallographic information; 3D view; checkCIF report

## Figures and Tables

**Table 1:** Hydrogen-bond geometry (Å, °)

*D*—H⋯*A*	*D*—H	H⋯*A*	*D*⋯*A*	*D*—H⋯*A*
O1*W*—H1⋯O12^i^	0.82 (9)	2.06 (9)	2.831 (11)	157 (9)
O11—H4⋯O1*W*	0.87 (11)	1.81 (11)	2.684 (13)	180 (1)

Symmetry code: (i) 
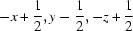
.

**Fig. 1.**
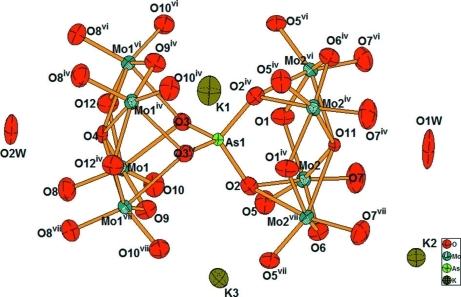
ORTEP drawing of the title compound withthermal ellipsoids at 50% probability.

**Fig. 2.**
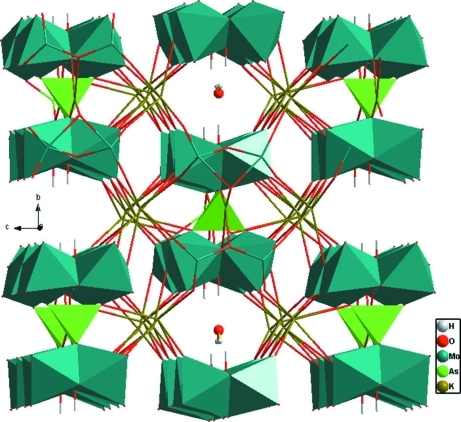
A polyhedral representationof the three-dimensional network structure of the title compound. All of the water molecular is omitted forclarity. [symmetry codes:(i) x, y,z; (ii) -x, -y, 1/2 + z; (iii) x, -y, -z; (iv) -x, y, 1/2 - z; (v) -x, -y, -z; (vi) x, y, 1/2 + z; (vii) -x, y, z; (viii) x, -y, 1/2 + z.]

## supplementary crystallographic information

### Comment

Polyoxometalates(POMs) are metal-oxygen anionic clusters, which act as multidentate inorganic ligands. They can bind most transition metals, rare earth metals and alkali metals, leading to a family of compounds with a variety of structures (Pope, 1983). Performing as an important part in this field, a number of compounds from the A/Mo/P/O system have been synthesized and structurally characterized, where A is an organic or inorganic cation. In contrast to the rich structural chemistry of molybdenum phosphates, the Mo/As/O system remains relatively undeveloped (Sun *et al.*, 2007; He & Wang, 1999). Here, we have hydrothermally synthesized the title compound, K_5_Mo_8_O_24_(OH)_2_AsO_4_.2H_2_O, which is isostructural with K_5-x_(NH_4_)_x_P[Mo_4_O_14_(OH)]_2_.2 H_2_O where x=2.43 (Chen *et al.* (2009).

The structure of the title compound consists of [AsMo_8_O_28_(OH)_2_]^5-^cluster anions, K^+^ cations, and water molecules (Fig. 1). The anion is formed by two crystallographically independent {Mo_4_O_14_(OH)}cores, linked by an arsenic(V) atom in a sandwich-like fashion. The anion possesses *m*2*m* symmetry in that all four molybdenum atoms in the {Mo_4_O_14_(OH)}core are coplanar, and one oxygen atom in each core is characterized as hydroxyl (O4 and O11) which is indicated by literature and bond valence sum calculation value (Hsu & Wang 1997, Brown *et al.*, 1981). The [AsMo_8_O_30_H_2_]^5-^cluster anions are bridged together by K^+^ cations into one-dimensional strings firstly. The anion-cation interactions are further extended into a three-dimensional architecture by other two K^+^ cations with each linking four cluster anions and one water molecule.

### Experimental

The title compound was synthesized by hydrothermal reaction of Fe(NO_3_)_3_.9 H_2_O(2.5 mmol),As_2_O_3 _(2.5 mmol), MoO_3_.2H_2_O (3.0 mmol),KOH (5.0 mmol) and H_2_O(18 ml) were stirred for 120 min. The pH of the mixture was adjusted to 6.5 with 1M nitric acid. The resultant mixture was sealed in a 25 ml Teflon-lined autoclave andheated at 180 *^o^*C for 8 d. The autoclave was then cooled to room temperature. The crystalline product was filtered, washed with distilled water and dried at ambient temperature to give black block solids.

### Refinement

The H atoms attached to oxygen molecular were located from difference Fourier maps, and the H atom attached o2w was disordered. The highest peak in the Fourier map is located 1.30Å from O9.

### Crystal data

**Table:**

K_5_[Mo_8_O_24_(OH)_2_(AsO_4_)]·2H_2_O	*F*(000) = 2904
*M**_r_* = 1553.95	*D*_x_ = 3.541 Mg m^−^^3^
Orthorhombic, *C**m**c**m*	Mo *K*α radiation, λ = 0.71073 Å
Hall symbol: -C 2c 2	Cell parameters from 3063 reflections
*a* = 8.3024 (12) Å	θ = 2.6–26.0°
*b* = 23.008 (3) Å	µ = 5.28 mm^−^^1^
*c* = 15.279 (2) Å	*T* = 293 K
*V* = 2918.6 (7) Å^3^	Block, black
*Z* = 4	0.20 × 0.18 × 0.16 mm

### Data collection

**Table:**

Rigaku R-AXIS RAPID diffractometer	1606 independent reflections
Radiation source: fine-focus sealed tube	1216 reflections with *I* > 2σ(*I*)
graphite	*R*_int_ = 0.048
Detector resolution: 10 pixels mm^-1^	θ_max_ = 26.0°, θ_min_ = 2.2°
ω scans	*h* = −10→10
Absorption correction: multi-scan (*ABSCOR*; Higashi, 1995)	*k* = −28→28
*T*_min_ = 0.418, *T*_max_ = 0.486	*l* = −18→18
12346 measured reflections	

### Refinement

**Table:**

Refinement on *F*^2^	Primary atom site location: structure-invariant direct methods
Least-squares matrix: full	Secondary atom site location: difference Fourier map
*R*[*F*^2^ > 2σ(*F*^2^)] = 0.032	Hydrogen site location: inferred from neighbouring sites
*wR*(*F*^2^) = 0.098	Only H-atom coordinates refined
*S* = 1.07	*w* = 1/[σ^2^(*F*_o_^2^) + (0.055*P*)^2^] where *P* = (*F*_o_^2^ + 2*F*_c_^2^)/3
1606 reflections	(Δ/σ)_max_ < 0.001
133 parameters	Δρ_max_ = 1.99 e Å^−^^3^
0 restraints	Δρ_min_ = −0.72 e Å^−^^3^

### Special details

**Table:**

Geometry. All esds (except the esd in the dihedral angle between two l.s. planes) are estimated using the full covariance matrix. The cell esds are taken into account individually in the estimation of esds in distances, angles and torsion angles; correlations between esds in cell parameters are only used when they are defined by crystal symmetry. An approximate (isotropic) treatment of cell esds is used for estimating esds involving l.s. planes.
Refinement. Refinement of *F*^2^ against ALL reflections. The weighted *R*-factor *wR* and goodness of fit *S* are based on *F*^2^, conventional *R*-factors *R* are based on *F*, with *F* set to zero for negative *F*^2^. The threshold expression of *F*^2^ > σ(*F*^2^) is used only for calculating *R*-factors(gt) *etc*. and is not relevant to the choice of reflections for refinement. *R*-factors based on *F*^2^ are statistically about twice as large as those based on *F*, and *R*- factors based on ALL data will be even larger.

### Fractional atomic coordinates and isotropic or equivalent isotropic displacement parameters (Å^2^)

**Table:**

	*x*	*y*	*z*	*U*_iso_*/*U*_eq_	Occ. (<1)
O2W	0.5000	0.3660 (4)	0.7500	0.046 (3)	
H2W	0.4128	0.3599	0.7224	0.069*	0.50
Mo1	0.30043 (6)	0.49010 (2)	0.35477 (3)	0.01904 (18)	
Mo2	0.30698 (6)	0.26333 (2)	0.35911 (3)	0.02029 (18)	
As1	0.5000	0.37665 (4)	0.2500	0.0140 (3)	
K1	0.0000	0.38267 (14)	0.2500	0.0504 (10)	
K2	0.5000	0.12103 (8)	0.46558 (14)	0.0300 (5)	
K3	0.5000	0.36453 (9)	0.52457 (14)	0.0341 (5)	
O12	0.1808 (7)	0.5103 (3)	0.2500	0.0332 (16)	
O11	0.5000	0.2312 (3)	0.2500	0.0198 (18)	
O10	0.1715 (6)	0.4466 (2)	0.4095 (3)	0.0379 (12)	
O9	0.5000	0.4605 (3)	0.3962 (4)	0.0328 (15)	
O8	0.2890 (6)	0.55608 (19)	0.4041 (3)	0.0404 (13)	
O7	0.2196 (7)	0.1971 (2)	0.3681 (3)	0.0516 (15)	
O6	0.5000	0.2443 (3)	0.4234 (4)	0.0341 (16)	
O5	0.2029 (6)	0.30763 (19)	0.4280 (3)	0.0382 (12)	
O4	0.5000	0.5206 (3)	0.2500	0.0189 (17)	
O3	0.3350 (7)	0.4194 (2)	0.2500	0.0268 (14)	
O2	0.5000	0.3333 (2)	0.3397 (4)	0.0288 (15)	
O1	0.2301 (8)	0.2928 (3)	0.2500	0.0338 (15)	
O1W	0.5000	0.1146 (4)	0.2500	0.067 (5)	
H1	0.426 (11)	0.091 (4)	0.2500	0.04 (3)*	
H4	0.5000	0.193 (5)	0.2500	0.02 (3)*	
H3	0.5000	0.562 (5)	0.2500	0.02 (3)*	

### Atomic displacement parameters (Å^2^)

**Table:**

	*U*^11^	*U*^22^	*U*^33^	*U*^12^	*U*^13^	*U*^23^
O2W	0.046 (7)	0.017 (5)	0.076 (8)	0.000	0.000	0.000
Mo1	0.0219 (3)	0.0168 (3)	0.0184 (3)	0.0015 (2)	0.0028 (2)	−0.00153 (19)
Mo2	0.0246 (4)	0.0173 (3)	0.0190 (3)	−0.0017 (2)	0.0040 (2)	0.00186 (19)
As1	0.0179 (6)	0.0104 (5)	0.0138 (6)	0.000	0.000	0.000
K1	0.0242 (18)	0.047 (2)	0.080 (3)	0.000	0.000	0.000
K2	0.0328 (12)	0.0281 (11)	0.0291 (11)	0.000	0.000	−0.0002 (8)
K3	0.0390 (13)	0.0324 (11)	0.0310 (11)	0.000	0.000	−0.0039 (9)
O12	0.027 (4)	0.039 (4)	0.034 (4)	0.006 (3)	0.000	0.000
O11	0.032 (5)	0.012 (4)	0.016 (4)	0.000	0.000	0.000
O10	0.036 (3)	0.035 (3)	0.042 (3)	−0.004 (2)	0.016 (2)	0.000 (2)
O9	0.029 (4)	0.038 (4)	0.031 (4)	0.000	0.000	0.005 (3)
O8	0.051 (4)	0.028 (2)	0.042 (3)	0.005 (2)	0.006 (3)	−0.006 (2)
O7	0.058 (4)	0.031 (3)	0.066 (4)	−0.013 (3)	0.021 (3)	0.006 (2)
O6	0.042 (4)	0.035 (4)	0.025 (3)	0.000	0.000	0.005 (3)
O5	0.046 (3)	0.034 (3)	0.034 (3)	0.002 (2)	0.021 (2)	0.000 (2)
O4	0.021 (5)	0.009 (4)	0.027 (4)	0.000	0.000	0.000
O3	0.030 (4)	0.025 (3)	0.025 (3)	−0.006 (3)	0.000	0.000
O2	0.032 (4)	0.027 (3)	0.027 (3)	0.000	0.000	−0.005 (3)
O1	0.029 (4)	0.047 (4)	0.026 (3)	0.003 (3)	0.000	0.000
O1W	0.039 (8)	0.012 (5)	0.150 (14)	0.000	0.000	0.000

### Geometric parameters (Å, °)

**Table:**

Mo1—O10	1.687 (4)	K2—O8^viii^	2.979 (5)
Mo1—O8	1.697 (4)	K2—O8^ix^	2.979 (5)
Mo1—O9	1.900 (3)	K2—O7^ii^	3.272 (6)
Mo1—O12	1.940 (3)	K2—O7	3.272 (6)
Mo1—O3	2.300 (4)	K2—O1W	3.297 (2)
Mo1—O4	2.409 (2)	K2—O1W	3.297 (2)
Mo1—Mo1^i^	3.2015 (10)	K2—Mo2^ii^	3.9915 (18)
Mo1—K1	3.859 (2)	K3—O8^x^	2.756 (5)
Mo2—O7	1.692 (5)	K3—O8^xi^	2.756 (5)
Mo2—O5	1.701 (4)	K3—O7^vi^	2.833 (5)
Mo2—O1	1.910 (3)	K3—O7^vii^	2.833 (5)
Mo2—O6	1.930 (3)	K3—O2	2.914 (6)
Mo2—O2	2.291 (4)	K3—O9	2.953 (6)
Mo2—O11	2.427 (2)	K3—O5	3.158 (5)
Mo2—Mo2^ii^	3.2050 (12)	K3—O5^ii^	3.158 (5)
Mo2—K3	3.792 (2)	K3—O6	3.168 (6)
Mo2—K2	3.9915 (18)	K3—Mo2^ii^	3.7922 (19)
Mo2—K1	4.100 (2)	K3—K2^xii^	4.1672 (6)
As1—O3^iii^	1.687 (6)	O12—Mo1^i^	1.940 (3)
As1—O3	1.687 (6)	O11—Mo2^ii^	2.427 (2)
As1—O2	1.695 (6)	O11—Mo2^i^	2.427 (2)
As1—O2^i^	1.695 (6)	O11—Mo2^iii^	2.427 (2)
K1—O1^iv^	2.815 (7)	O10—K2^vii^	2.844 (5)
K1—O1	2.815 (7)	O9—Mo1^ii^	1.900 (3)
K1—O3	2.907 (6)	O8—K3^x^	2.756 (5)
K1—O3^iv^	2.907 (6)	O8—K2^xiii^	2.979 (5)
K1—O10	3.183 (5)	O7—K3^vii^	2.833 (5)
K1—O10^v^	3.183 (5)	O6—Mo2^ii^	1.930 (3)
K1—O10^iv^	3.183 (5)	O5—K2^vii^	2.859 (4)
K1—O10^i^	3.183 (5)	O4—Mo1^i^	2.409 (2)
K1—O12^iv^	3.298 (7)	O4—Mo1^ii^	2.409 (2)
K1—O12	3.298 (7)	O4—Mo1^iii^	2.409 (2)
K1—Mo1^i^	3.859 (2)	O3—Mo1^i^	2.300 (4)
K2—O10^vi^	2.844 (5)	O2—Mo2^ii^	2.291 (4)
K2—O10^vii^	2.844 (5)	O1—Mo2^i^	1.910 (3)
K2—O5^vi^	2.859 (4)	O1W—O1W	0.00 (2)
K2—O5^vii^	2.859 (4)	O1W—K2^i^	3.297 (2)
K2—O6	2.909 (6)		
			
O10—Mo1—O8	106.0 (2)	O10^vi^—K2—O7^ii^	104.00 (13)
O10—Mo1—O9	100.1 (2)	O10^vii^—K2—O7^ii^	162.64 (14)
O8—Mo1—O9	102.8 (3)	O5^vi^—K2—O7^ii^	62.12 (14)
O10—Mo1—O12	103.1 (2)	O5^vii^—K2—O7^ii^	111.77 (14)
O8—Mo1—O12	97.1 (2)	O6—K2—O7^ii^	51.49 (10)
O9—Mo1—O12	143.8 (2)	O8^viii^—K2—O7^ii^	134.27 (14)
O10—Mo1—O3	90.3 (2)	O8^ix^—K2—O7^ii^	63.37 (14)
O8—Mo1—O3	161.5 (2)	O10^vi^—K2—O7	162.64 (14)
O9—Mo1—O3	82.5 (2)	O10^vii^—K2—O7	104.00 (13)
O12—Mo1—O3	70.04 (19)	O5^vi^—K2—O7	111.77 (14)
O10—Mo1—O4	159.8 (2)	O5^vii^—K2—O7	62.12 (13)
O8—Mo1—O4	94.2 (2)	O6—K2—O7	51.49 (10)
O9—Mo1—O4	74.11 (18)	O8^viii^—K2—O7	63.37 (13)
O12—Mo1—O4	74.57 (16)	O8^ix^—K2—O7	134.27 (14)
O3—Mo1—O4	70.0 (2)	O7^ii^—K2—O7	90.72 (18)
O10—Mo1—Mo1^i^	119.71 (17)	O10^vi^—K2—O1W	130.22 (18)
O8—Mo1—Mo1^i^	116.36 (16)	O10^vii^—K2—O1W	130.22 (18)
O9—Mo1—Mo1^i^	109.46 (18)	O5^vi^—K2—O1W	126.43 (16)
O12—Mo1—Mo1^i^	34.40 (15)	O5^vii^—K2—O1W	126.43 (16)
O3—Mo1—Mo1^i^	45.89 (10)	O6—K2—O1W	79.8 (2)
O4—Mo1—Mo1^i^	48.35 (5)	O8^viii^—K2—O1W	70.27 (14)
O10—Mo1—K1	54.24 (16)	O8^ix^—K2—O1W	70.27 (14)
O8—Mo1—K1	136.12 (19)	O7^ii^—K2—O1W	64.48 (14)
O9—Mo1—K1	118.16 (19)	O7—K2—O1W	64.48 (14)
O12—Mo1—K1	58.68 (19)	O10^vi^—K2—O1W	130.22 (18)
O3—Mo1—K1	48.63 (15)	O10^vii^—K2—O1W	130.22 (18)
O4—Mo1—K1	110.84 (10)	O5^vi^—K2—O1W	126.43 (16)
Mo1^i^—Mo1—K1	65.495 (16)	O5^vii^—K2—O1W	126.43 (16)
O7—Mo2—O5	105.7 (2)	O6—K2—O1W	79.8 (2)
O7—Mo2—O1	104.3 (3)	O8^viii^—K2—O1W	70.27 (14)
O5—Mo2—O1	99.1 (2)	O8^ix^—K2—O1W	70.27 (14)
O7—Mo2—O6	96.4 (3)	O7^ii^—K2—O1W	64.48 (14)
O5—Mo2—O6	104.0 (2)	O7—K2—O1W	64.48 (14)
O1—Mo2—O6	143.4 (2)	O1W—K2—O1W	0.0 (4)
O7—Mo2—O2	160.5 (2)	O10^vi^—K2—Mo2^ii^	121.40 (10)
O5—Mo2—O2	90.80 (19)	O10^vii^—K2—Mo2^ii^	157.37 (11)
O1—Mo2—O2	82.6 (2)	O5^vi^—K2—Mo2^ii^	61.60 (9)
O6—Mo2—O2	69.15 (19)	O5^vii^—K2—Mo2^ii^	89.85 (10)
O7—Mo2—O11	93.7 (2)	O6—K2—Mo2^ii^	27.12 (6)
O5—Mo2—O11	160.5 (2)	O8^viii^—K2—Mo2^ii^	127.33 (10)
O1—Mo2—O11	74.28 (19)	O8^ix^—K2—Mo2^ii^	87.68 (9)
O6—Mo2—O11	74.49 (16)	O7^ii^—K2—Mo2^ii^	24.47 (9)
O2—Mo2—O11	70.33 (19)	O7—K2—Mo2^ii^	70.20 (9)
O7—Mo2—Mo2^ii^	115.4 (2)	O1W—K2—Mo2^ii^	68.26 (16)
O5—Mo2—Mo2^ii^	120.53 (17)	O1W—K2—Mo2^ii^	68.26 (16)
O1—Mo2—Mo2^ii^	109.53 (19)	O8^x^—K3—O8^xi^	78.9 (2)
O6—Mo2—Mo2^ii^	33.86 (14)	O8^x^—K3—O7^vi^	72.18 (16)
O2—Mo2—Mo2^ii^	45.62 (10)	O8^xi^—K3—O7^vi^	120.80 (17)
O11—Mo2—Mo2^ii^	48.69 (5)	O8^x^—K3—O7^vii^	120.80 (17)
O7—Mo2—K3	132.82 (18)	O8^xi^—K3—O7^vii^	72.18 (16)
O5—Mo2—K3	55.55 (17)	O7^vi^—K3—O7^vii^	80.1 (2)
O1—Mo2—K3	120.34 (19)	O8^x^—K3—O2	123.14 (14)
O6—Mo2—K3	56.54 (18)	O8^xi^—K3—O2	123.14 (14)
O2—Mo2—K3	50.13 (14)	O7^vi^—K3—O2	115.96 (14)
O11—Mo2—K3	111.44 (10)	O7^vii^—K3—O2	115.96 (14)
Mo2^ii^—Mo2—K3	65.002 (16)	O8^x^—K3—O9	76.54 (14)
O7—Mo2—K2	53.2 (2)	O8^xi^—K3—O9	76.54 (14)
O5—Mo2—K2	116.31 (15)	O7^vi^—K3—O9	139.37 (12)
O1—Mo2—K2	141.38 (18)	O7^vii^—K3—O9	139.37 (12)
O6—Mo2—K2	43.40 (17)	O2—K3—O9	62.64 (17)
O2—Mo2—K2	110.39 (11)	O8^x^—K3—O5	162.95 (14)
O11—Mo2—K2	76.43 (14)	O8^xi^—K3—O5	87.88 (13)
Mo2^ii^—Mo2—K2	66.329 (14)	O7^vi^—K3—O5	124.43 (15)
K3—Mo2—K2	93.57 (4)	O7^vii^—K3—O5	63.91 (14)
O7—Mo2—K1	111.7 (2)	O2—K3—O5	56.30 (9)
O5—Mo2—K1	62.27 (16)	O9—K3—O5	89.98 (12)
O1—Mo2—K1	36.82 (17)	O8^x^—K3—O5^ii^	87.88 (13)
O6—Mo2—K1	151.01 (18)	O8^xi^—K3—O5^ii^	162.95 (14)
O2—Mo2—K1	84.92 (11)	O7^vi^—K3—O5^ii^	63.91 (14)
O11—Mo2—K1	109.56 (11)	O7^vii^—K3—O5^ii^	124.43 (15)
Mo2^ii^—Mo2—K1	128.43 (3)	O2—K3—O5^ii^	56.30 (9)
K3—Mo2—K1	97.04 (4)	O9—K3—O5^ii^	89.98 (12)
K2—Mo2—K1	164.67 (4)	O5—K3—O5^ii^	102.71 (16)
O3^iii^—As1—O3	108.6 (4)	O8^x^—K3—O6	140.50 (10)
O3^iii^—As1—O2	110.06 (13)	O8^xi^—K3—O6	140.50 (10)
O3—As1—O2	110.06 (13)	O7^vi^—K3—O6	81.10 (14)
O3^iii^—As1—O2^i^	110.06 (13)	O7^vii^—K3—O6	81.10 (14)
O3—As1—O2^i^	110.06 (13)	O2—K3—O6	46.54 (15)
O2—As1—O2^i^	108.0 (4)	O9—K3—O6	109.18 (17)
O1^iv^—K1—O1	85.5 (3)	O5—K3—O6	53.87 (9)
O1^iv^—K1—O3	149.7 (2)	O5^ii^—K3—O6	53.87 (9)
O1—K1—O3	64.18 (17)	O8^x^—K3—Mo2	159.94 (12)
O1^iv^—K1—O3^iv^	64.18 (17)	O8^xi^—K3—Mo2	113.71 (10)
O1—K1—O3^iv^	149.7 (2)	O7^vi^—K3—Mo2	110.51 (12)
O3—K1—O3^iv^	146.2 (3)	O7^vii^—K3—Mo2	78.84 (11)
O1^iv^—K1—O10	130.02 (9)	O2—K3—Mo2	37.12 (8)
O1—K1—O10	92.05 (13)	O9—K3—Mo2	90.93 (12)
O3—K1—O10	55.77 (9)	O5—K3—Mo2	26.37 (8)
O3^iv^—K1—O10	107.08 (13)	O5^ii^—K3—Mo2	76.36 (9)
O1^iv^—K1—O10^v^	92.05 (13)	O6—K3—Mo2	30.55 (6)
O1—K1—O10^v^	130.02 (9)	O8^x^—K3—Mo2^ii^	113.71 (10)
O3—K1—O10^v^	107.08 (13)	O8^xi^—K3—Mo2^ii^	159.94 (12)
O3^iv^—K1—O10^v^	55.77 (9)	O7^vi^—K3—Mo2^ii^	78.84 (11)
O10—K1—O10^v^	53.16 (17)	O7^vii^—K3—Mo2^ii^	110.51 (12)
O1^iv^—K1—O10^iv^	92.05 (13)	O2—K3—Mo2^ii^	37.12 (8)
O1—K1—O10^iv^	130.02 (9)	O9—K3—Mo2^ii^	90.93 (12)
O3—K1—O10^iv^	107.08 (13)	O5—K3—Mo2^ii^	76.36 (9)
O3^iv^—K1—O10^iv^	55.77 (9)	O5^ii^—K3—Mo2^ii^	26.37 (8)
O10—K1—O10^iv^	125.0 (2)	O6—K3—Mo2^ii^	30.55 (6)
O10^v^—K1—O10^iv^	99.94 (17)	Mo2—K3—Mo2^ii^	50.00 (3)
O1^iv^—K1—O10^i^	130.02 (9)	O8^x^—K3—K2^xii^	45.54 (10)
O1—K1—O10^i^	92.05 (13)	O8^xi^—K3—K2^xii^	124.48 (12)
O3—K1—O10^i^	55.77 (9)	O7^vi^—K3—K2^xii^	51.54 (12)
O3^iv^—K1—O10^i^	107.08 (13)	O7^vii^—K3—K2^xii^	131.30 (14)
O10—K1—O10^i^	99.94 (17)	O2—K3—K2^xii^	93.13 (4)
O10^v^—K1—O10^i^	125.0 (2)	O9—K3—K2^xii^	87.96 (4)
O10^iv^—K1—O10^i^	53.16 (17)	O5—K3—K2^xii^	145.89 (10)
O1^iv^—K1—O12^iv^	110.18 (16)	O5^ii^—K3—K2^xii^	43.27 (8)
O1—K1—O12^iv^	164.35 (19)	O6—K3—K2^xii^	95.00 (4)
O3—K1—O12^iv^	100.17 (18)	Mo2—K3—K2^xii^	119.60 (5)
O3^iv^—K1—O12^iv^	46.00 (15)	Mo2^ii^—K3—K2^xii^	69.64 (3)
O10—K1—O12^iv^	78.02 (13)	Mo1—O12—Mo1^i^	111.2 (3)
O10^v^—K1—O12^iv^	52.04 (9)	Mo1—O12—K1	91.2 (2)
O10^iv^—K1—O12^iv^	52.04 (9)	Mo1^i^—O12—K1	91.2 (2)
O10^i^—K1—O12^iv^	78.02 (13)	Mo2^ii^—O11—Mo2^i^	144.6 (3)
O1^iv^—K1—O12	164.35 (19)	Mo2^ii^—O11—Mo2^iii^	86.75 (11)
O1—K1—O12	110.18 (16)	Mo2^i^—O11—Mo2^iii^	82.63 (10)
O3—K1—O12	46.00 (15)	Mo2^ii^—O11—Mo2	82.63 (10)
O3^iv^—K1—O12	100.17 (18)	Mo2^i^—O11—Mo2	86.75 (11)
O10—K1—O12	52.04 (9)	Mo2^iii^—O11—Mo2	144.6 (3)
O10^v^—K1—O12	78.02 (13)	Mo1—O10—K2^vii^	167.1 (3)
O10^iv^—K1—O12	78.02 (13)	Mo1—O10—K1	100.3 (2)
O10^i^—K1—O12	52.04 (9)	K2^vii^—O10—K1	92.12 (14)
O12^iv^—K1—O12	54.2 (2)	Mo1^ii^—O9—Mo1	121.4 (3)
O1^iv^—K1—Mo1	155.37 (3)	Mo1^ii^—O9—K3	119.31 (16)
O1—K1—Mo1	91.82 (12)	Mo1—O9—K3	119.31 (16)
O3—K1—Mo1	36.42 (8)	Mo1—O8—K3^x^	137.1 (3)
O3^iv^—K1—Mo1	115.59 (13)	Mo1—O8—K2^xiii^	129.3 (3)
O10—K1—Mo1	25.48 (8)	K3^x^—O8—K2^xiii^	93.14 (13)
O10^v^—K1—Mo1	71.07 (10)	Mo2—O7—K3^vii^	140.7 (3)
O10^iv^—K1—Mo1	108.11 (12)	Mo2—O7—K2	102.3 (3)
O10^i^—K1—Mo1	74.48 (9)	K3^vii^—O7—K2	85.77 (13)
O12^iv^—K1—Mo1	73.98 (11)	Mo2^ii^—O6—Mo2	112.3 (3)
O12—K1—Mo1	30.17 (6)	Mo2^ii^—O6—K2	109.48 (19)
O1^iv^—K1—Mo1^i^	155.37 (3)	Mo2—O6—K2	109.48 (19)
O1—K1—Mo1^i^	91.82 (12)	Mo2^ii^—O6—K3	92.91 (19)
O3—K1—Mo1^i^	36.42 (8)	Mo2—O6—K3	92.91 (19)
O3^iv^—K1—Mo1^i^	115.59 (13)	K2—O6—K3	138.0 (2)
O10—K1—Mo1^i^	74.48 (9)	Mo2—O5—K2^vii^	174.3 (3)
O10^v^—K1—Mo1^i^	108.11 (12)	Mo2—O5—K3	98.1 (2)
O10^iv^—K1—Mo1^i^	71.07 (10)	K2^vii^—O5—K3	87.51 (12)
O10^i^—K1—Mo1^i^	25.48 (8)	Mo1^i^—O4—Mo1^ii^	146.1 (3)
O12^iv^—K1—Mo1^i^	73.98 (11)	Mo1^i^—O4—Mo1	83.31 (9)
O12—K1—Mo1^i^	30.17 (6)	Mo1^ii^—O4—Mo1	86.93 (10)
Mo1—K1—Mo1^i^	49.01 (3)	Mo1^i^—O4—Mo1^iii^	86.93 (10)
O10^vi^—K2—O10^vii^	60.1 (2)	Mo1^ii^—O4—Mo1^iii^	83.31 (9)
O10^vi^—K2—O5^vi^	68.74 (15)	Mo1—O4—Mo1^iii^	146.1 (3)
O10^vii^—K2—O5^vi^	103.14 (16)	As1—O3—Mo1^i^	120.9 (2)
O10^vi^—K2—O5^vii^	103.14 (16)	As1—O3—Mo1	120.9 (2)
O10^vii^—K2—O5^vii^	68.74 (15)	Mo1^i^—O3—Mo1	88.2 (2)
O5^vi^—K2—O5^vii^	72.2 (2)	As1—O3—K1	127.4 (3)
O10^vi^—K2—O6	132.99 (14)	Mo1^i^—O3—K1	94.95 (18)
O10^vii^—K2—O6	132.99 (14)	Mo1—O3—K1	94.95 (18)
O5^vi^—K2—O6	64.28 (13)	As1—O2—Mo2	121.18 (19)
O5^vii^—K2—O6	64.28 (13)	As1—O2—Mo2^ii^	121.18 (19)
O10^vi^—K2—O8^viii^	109.91 (14)	Mo2—O2—Mo2^ii^	88.8 (2)
O10^vii^—K2—O8^viii^	62.22 (14)	As1—O2—K3	129.7 (3)
O5^vi^—K2—O8^viii^	160.37 (15)	Mo2—O2—K3	92.75 (17)
O5^vii^—K2—O8^viii^	89.59 (13)	Mo2^ii^—O2—K3	92.75 (17)
O6—K2—O8^viii^	114.80 (11)	Mo2—O1—Mo2^i^	121.6 (3)
O10^vi^—K2—O8^ix^	62.22 (14)	Mo2—O1—K1	119.19 (17)
O10^vii^—K2—O8^ix^	109.91 (14)	Mo2^i^—O1—K1	119.19 (17)
O5^vi^—K2—O8^ix^	89.59 (13)	O1W—O1W—K2^i^	0(10)
O5^vii^—K2—O8^ix^	160.37 (15)	O1W—O1W—K2	0(10)
O6—K2—O8^ix^	114.80 (11)	K2^i^—O1W—K2	174.9 (4)
O8^viii^—K2—O8^ix^	107.32 (19)		

Symmetry codes: (i) *x*, *y*, −*z*+1/2; (ii) −*x*+1, *y*, *z*; (iii) −*x*+1, *y*, −*z*+1/2; (iv) −*x*, *y*, −*z*+1/2; (v) −*x*, *y*, *z*; (vi) *x*+1/2, −*y*+1/2, −*z*+1; (vii) −*x*+1/2, −*y*+1/2, −*z*+1; (viii) −*x*+1/2, *y*−1/2, *z*; (ix) *x*+1/2, *y*−1/2, *z*;  (x) −*x*+1, −*y*+1, −*z*+1; (xi) *x*, −*y*+1, −*z*+1; (xii) −*x*+3/2, −*y*+1/2, −*z*+1; (xiii) *x*−1/2, *y*+1/2, *z*.

### Hydrogen-bond geometry (Å, °)

**Table:**

*D*—H···*A*	*D*—H	H···*A*	*D*···*A*	*D*—H···*A*
O1W—H1···O12^xiv^	0.82 (9)	2.06 (9)	2.831 (11)	157 (9)
O11—H4···O1W	0.87 (11)	1.81 (11)	2.684 (13)	180.(1)

Symmetry codes: (xiv) −*x*+1/2, *y*−1/2, −*z*+1/2.
